# Musical training, bilingualism, and executive function: working memory and inhibitory control

**DOI:** 10.1186/s41235-018-0095-6

**Published:** 2018-04-11

**Authors:** Annalise A. D’Souza, Linda Moradzadeh, Melody Wiseheart

**Affiliations:** 1 Department of Psychology, York University, 4700 Keele Street, Toronto, ON M3J 1P3 Canada; 2 LaMarsh Centre for Child and Youth Research, York University, Toronto, ON Canada

**Keywords:** Bilingualism, Executive function, Inhibition, Music, Training, Working memory

## Abstract

The current study investigated whether long-term experience in music or a second language is associated with enhanced cognitive functioning. Early studies suggested the possibility of a cognitive advantage from musical training and bilingualism but have failed to be replicated by recent findings. Further, each form of expertise has been independently investigated leaving it unclear whether any benefits are specifically caused by each skill or are a result of skill learning in general.

To assess whether cognitive benefits from training exist, and how unique they are to each training domain, the current study compared musicians and bilinguals to each other, plus to individuals who had expertise in both skills, or neither. Young adults (*n* = 153) were categorized into one of four groups: monolingual musician; bilingual musician; bilingual non-musician; and monolingual non-musician. Multiple tasks per cognitive ability were used to examine the coherency of any training effects. Results revealed that musically trained individuals, but not bilinguals, had enhanced working memory. Neither skill had enhanced inhibitory control.

The findings confirm previous associations between musicians and improved cognition and extend existing evidence to show that benefits are narrower than expected but can be uniquely attributed to music compared to another specialized auditory skill domain. The null bilingual effect despite a music effect in the same group of individuals challenges the proposition that young adults are at a performance ceiling and adds to increasing evidence on the lack of a bilingual advantage on cognition.

## Significance

The idea that everyday activities such as learning music or speaking a second language can make a person smarter has gained increasing popularity. The good news is that early research studies found better mental performance in musicians and bilinguals. However, recent investigations failed to reproduce performance improvements in experts, leaving it uncertain whether robust benefits exist. Moreover, since musical training and bilingualism have largely been tested separately, the relative benefits from each remain unclear. Before findings can be applied to real-world settings such as education, healthy development and aging, and cognitive dysfunction, the reliability and uniqueness of a cognitive advantage in experts must be established.

To address this need, the current study compared groups of people who were experts in music, bilingualism, both, or none. We tested whether experts outperformed their non-trained counterparts on cognition and whether any benefits were specific to distinct cognitive abilities (working memory vs interference control) and skills (music vs bilingualism). Multiple tasks were used to confirm if effects were coherent across measures of a cognitive ability.

Results showed that musically trained individuals outperformed their non-trained counterparts on the ability to maintain and manipulate information across measures, but not on the ability to resist distracting information. No mental enhancements were observed from bilingualism or from having both skills (being a bilingual musician), demonstrating that training effects are much more limited than previous evidence suggests. Our findings show that augmented mental performance in skill experts is possible but limited to only some skills and some mental abilities.

## Background

The idea that learning to play music or speak a second language can make a person smarter has gained increasing attention and popularity in public spheres. In line with this notion, research findings support that musicians can demonstrate better cognitive performance than non-musicians and similarly for bilinguals compared with monolinguals (Bialystok, Hawrylewicz, Wiseheart, & Toplak, 2017; Schellenberg, 2016; Wiseheart, Viswanathan, & Bialystok, 2016). However, the consistency of such demonstrations is unclear. Recent studies have failed to replicate either a musician or bilingual advantage across different cognitive abilities or even tasks of the same ability, calling into question the reliability of any benefits (Paap & Sawi, 2014; Paap, Johnson, and Sawi, 2015; Sala & Gobet, 2017).

Even if benefits do exist, it is unclear whether such benefits are due to music or language itself. Since both musicians and bilinguals have shown cognitive enhancements, the simplest explanation may be that benefits are a general result of skill learning or expertise rather than being specific to a single skill. To investigate the uniqueness of the purported link of each skill with cognition, we directly compared musical training and bilingualism to assess their relative benefits. The existing evidence has investigated each form of expertise independently and produced inconsistent effects; hence, we were interested in whether enhancements could be consistently demonstrated and were unique to each skill.

The current study compared both skills on the cognitive abilities of working memory and inhibitory control. Working memory is the mechanism for the active manipulation and storage of temporary information (Baddeley, 2003; Baddeley & Hitch, 1974). Inhibitory control involves the active suppression of an automatic or “prepotent” response or thought and is a superset of interference control and response inhibition. Working memory and inhibitory control are types of executive functions, higher-order cognitive functions involved in goal-directed behavior (Diamond, 2013; Miyake, Friedman, Emerson, Witzki, Howerter, et al., 2000).

### Musical training, working memory, and inhibitory control

A body of evidence demonstrates that musicians outperform non-musicians on various cognitive abilities far beyond what the skill involves, such as verbal memory and visuospatial abilities (Costa-Giomi, 1999; Franklin et al., 2008). Beyond specific abilities, musicians have also demonstrated an advantage on broad cognitive abilities such as executive functions. Research evidence supports a strong association between enhanced working memory performance in musicians compared to non-musicians. Improved working memory was found across age groups, including children (Degé, Kubicek, & Schwarzer, 2011; c.f. Fujioka, Ross, Kakigi, Pantev, & Trainor, 2006; Lee, Lu, & Ko, 2007; Moreno et al., 2011; Roden, Kreutz, & Bongard, 2012; Schellenberg, 2011), young adults (Franklin et al., 2008; Meinz & Hambrick, 2010; c.f. Nutley, Darki, & Klingberg, 2014; Oechslin, Van de Ville, Lazeyras, Hauert, & James, 2013; Parbery-Clark, Skoe, Lam, & Kraus, 2009; Strait, Kraus, Parbery-Clark, & Ashley, 2010; Wallentin, Nielsen, Friis-Olivarius, Vuust, & Vuust, 2010; Zuk, Benjamin, Kenyon, & Gaab, 2014), and older adults (Amer, Kalender, Hasher, Trehub, & Wong, 2013; Bugos, Perlstein, McCrae, Brophy, & Bedenbaugh, 2007; Hanna-Pladdy & Gajewski, 2012; Hanna-Pladdy & MacKay, 2011).

Improvements have been found across different measures of working memory, such as simple and complex tasks (the former of which measure memory span or storage, and the latter which recruit additional executive control; Unsworth & Engle, 2006), plus for auditory and visuospatial domains, although there is some inconsistency in findings (Hanna-Pladdy & MacKay, 2011; Hansen, Wallentin, & Vuust, 2013; Lee et al., 2007; Strait et al., 2010). There is also evidence of better inhibitory control in musicians across age groups – in children (Degé et al., 2011; Moreno et al., 2011), young adults (Bialystok & Depape, 2009; Hurwitz, Wolff, Bortnick, & Kokas, 1975; Strait et al., 2010; Travis, Harung, & Lagrosen, 2011), and older adults (Seinfeld, Figueroa, Ortiz-Gil, & Sanchez-Vives, 2013). However, it is not consistently replicated across tasks or studies (Carey et al., 2015; Slevc, Davey, Buschkuehl, & Jaeggi, 2016; Zuk et al., 2014).

### Bilingualism, working memory, and inhibitory control

As with musical training, speaking a second language is associated with cognitive improvements outside the skill domain itself. Bilinguals outperform their monolingual counterparts in areas such as metalinguistic abilities and awareness of the mental states of others (Goetz, 2003; Gold, Kim, Johnson, Kryscio, & Smith, 2013). On general cognitive ability, early evidence suggested that bilinguals sometimes outperform monolinguals on non-linguistic interference paradigms, such as the Simon, Stroop, and flanker tasks (Bialystok, Craik, & Luk, 2008; Meuter & Allport, 1999). However, recent investigations using multiple tasks have failed to replicate the bilingual advantage, challenging the consistency of a bilingual cognitive advantage (Kousaie & Phillips, 2012a; Kousaie & Phillips, 2012b; Paap & Greenberg, 2013; Prior, 2012; von Bastian, Souza, & Gade, 2016).

Similarly, although some evidence supports a bilingual advantage in working memory (Bialystok, 2009; Bialystok & Feng, 2009; Luo, Craik, Moreno, & Bialystok, 2013), other studies have challenged this finding (Adesope, Lavin, Thompson, & Ungerleider, 2010; Bonifacci, Giombini, Bellocchi, & Contento, 2011; Feng, Diamond, & Bialystok, 2007; Ratiu & Azuma, 2015; von Bastian et al., 2016). It has been suggested that language effects exist for certain experimental conditions and populations but not others (Bialystok et al., 2017), but the finding of a bilingual effect has not consistently been replicated in other age groups (De Bruin, Treccani, & Della Sala, 2015; Gold et al., 2013, Exp. 2; Kousaie & Phillips, 2012a; Salvatierra & Rosselli, 2010). The overall findings from the bilingualism literature typically demonstrate only a few studies with large interference effects and a bilingual advantage and a majority of studies with smaller effects and little or no bilingual advantage (Duñabeitia et al., 2014). Reviews of bilingual evidence indicate that 80% of the literature yields null effects, with a greater percentage of null results for sample sizes larger than 40 (Paap, Johnson, & Sawi, 2015), in contrast with music where the evidence indicates a small to medium effect size (Sala & Gobet, 2017).

### Theoretical mechanisms for benefits from musical training and bilingualism

Independent demonstrations of cognitive associations with the domains of music and bilingualism open up the interesting research question of overlapping mechanisms between these domains that may produce cognitive benefits. The central metaphor is that both skills involve coordination of multiple abilities and domains of performance, and the regular exposure to a context where higher-level cognitive function is constantly in demand may contribute to advanced cognitive performance in experts. Higher-level cognition is theorized to be required for both skills, such as working memory to carry out maintaining and manipulating semantic information (e.g. notes, mechanics, rhythms in music; words, syntax, and timing in language) and inhibitory control in blocking or ignoring competing information internally or from the environment (e.g. external noise, irrelevant notes or words). It is plausible that expert musicians or language speakers, both of whom typically have spent at least ten years practicing their skills, demonstrate enhanced cognition compared to individuals without such training. The need for cognitive functions has been proposed via the executive function hypothesis in musicians (Hannon & Trainor, 2007) and the inhibitory control model in bilinguals (Bialystok, 2009), which later gave way to a more general global cognitive model (Bialystok, 2011).

Theoretical similarities in the processing resources for music and language have been proposed, since both are primarily auditory domains and overlap in organization of syntax and temporal structure (Patel, 2011, 2014; Slevc, 2012; Slevc & Okada, 2015). Indication of shared resources is seen in some bidirectionality between both skill domains; however, transfer from music to language abilities tends to be stronger and more widely supported than in the other direction (c.f. Asaridou & McQueen, 2013; Besson, Chobert, & Marie, 2011). Modularity, that is, separate processing in music, is also supported by evidence from musically impaired individuals, speech perception, and neuroscientific findings (Johansson, 2008; Mok & Zuo, 2012; Peretz, 2006). While both skill forms are cognitively demanding, they have different experience-specific demands (Hutka, Bidelman, & Moreno, 2013; Rogalsky, Rong, Saberi, & Hickok, 2011).

Theories on generalizability of learning or transfer of training support that effects of a given skill depend on the functional demands of learning that skill (Barnett & Ceci, Hickok, 2002; Morris, Bransford, & Franks, 1977). The closer a match between the content and context of a skill domain and a tested ability, the greater the effect observed. The role of similarity in determining cognitive benefits has been supported from evidence on cognition-perception couplings in music, linking aspects of the trained and transfer domains (Carey et al., 2015; Parbery-Clark et al., 2009).

Evidence from direct comparisons between language and music on cognitive abilities indicate that there are domain-specific effects that go beyond the shared processing between domains. Differential effects of each are seen on inhibitory control. Although musicians and bilinguals outperform monolingual musicians on some conflict resolution tasks, musicians showed better performance across tasks (Bialystok & DePape, 2009; Moreno, Wodniecka, Tays, Alain, & Bialystok, 2014). Similarly, music and language conferred benefits for some working memory tasks, but musicians had better performance across tasks (Bidelman, Hutka, & Moreno, 2013; Fiveash & Pammer, 2014).

### Current study

In light of recent evidence demonstrating training-induced changes in cognition, the present study investigated the degree to which musical training and bilingualism are associated with enhancements in working memory and inhibitory control. Overall, the current evidence indicates a strong association between musical training and working memory, a possible association with inhibitory control, and limited evidence for a bilingual benefit on either ability. However, several methodological issues preclude causal conclusions from the existing evidence, leaving two main questions: first, which abilities are influenced (if any); and, second, whether music or bilingualism uniquely produce benefits.

Different studies have used different tasks for a given construct, which has led to disparities in findings. Comprehensive investigations using multiple measures per construct have also not shown a consistent bilingual effect across tasks, suggesting that any previously demonstrated benefits may have been task-specific (Duñabeitia et al., 2014; Kousaie & Phillips, 2012a; Paap & Greenberg, 2013). To address this issue and resolve uncertainty in the literature, the present study included multiple tasks that measure each of our constructs of interest (working memory and inhibitory control). A coherent advantage would be demonstrated if there were benefits across several measures of the same construct, rather than a benefit limited to one specific measure.

A second issue arises since the majority of the existing evidence consists of non-experimental investigations comparing groups of skilled experts to non-experts. However, several non-musical and non-language background factors also exist between these groups and could be responsible for some of the observed effects. The largest confounding factors are socioeconomic status (SES) in the bilingual literature and intelligence in the music literature (Corrigall, Schellenberg, & Misura, 2013; Degé et al., 2011; Schellenberg, 2011). Since SES and intelligence are correlated with both our independent and dependent measures, it raises the issue of reverse causality in demonstrated effects (Valian, 2015). Such confounding factors can produce false advantages, or even hide true effects, yet are rarely accounted for in previous investigations (Schellenberg, 2016; Swaminathan & Schellenberg, 2014). To isolate unique effects of musical training and bilingualism, the current study used a sample that was homogenous in educational background and geographical location; and we tested for differences in possible third factors that are known to impact cognitive performance. Most studies have failed to find an effect of bilingualism above that of background variables (Antón et al., 2014; c.f. Calvo & Bialystok, 2014; Mindt et al., 2008; Morton & Harper, 2007; Ratiu & Azuma, 2015), although the evidence from musical training is more favorable (Degé et al., 2011).

To parse out effects unique to musical training, experimental trials have been conducted that randomly allocate untrained individuals who are matched on background factors to either musical training or a different group. Evidence from these trials shows little to no evidence of cognitive benefits of musical training on working memory or interference control (Mehr, Schachner, Katz, & Spelke, 2013; Moreno et al., 2011; Sala & Gobet, 2017). While experimental designs indicate that the small range of benefits observed can be attributed to musical training, they use a short period of training (several weeks to a year) and often an artificial setting. Thus, they address the reverse causality issue but are not comparable to correlational evidence which defines musicians as individuals who have received several years of training and use real-world musical training. Longitudinal designs over several years would address this issue but are practically difficult. More feasible would be correlational studies that control for prognostic variables that predict cognition (non-verbal intelligence, vocabulary, and SES) plus training factors to isolate training-induced effects. Hence, our study compares individuals with a long period of real-life training, while accounting for background and training factors (comparing between those with musical experience and bilingual experience) to address the specificity issue.

There were several predictions for the current study. First, we expected musicians to outperform non-musicians on working memory ability and inhibitory control. In terms of working memory, we expected musicians to have better accuracy than non-musicians on both executive and phonological working memory tasks, because musical training involves manipulation and maintenance of multiple symbols and codes. In terms of inhibitory control, we expected musicians to have faster response times on tasks that require suppression of interfering stimuli, as well as on tasks that require stopping an automatic response, because musical training involves ignoring irrelevant symbols and sounds as well as suddenly pausing and restarting performance when required, particularly in group musical performance.

Second, we expected bilinguals would outperform monolinguals on working memory ability and inhibitory control. As for musical training, it can be argued that bilingualism involves mentally manipulating different symbolic systems. However, the lack of replication in recent studies suggests that there is no coherent evidence for a bilingual inhibitory advantage. Newer explanations propose that a bilingual advantage may be either restricted to certain task conditions or a result of non-language background factors. Our study is especially relevant in light of recent work questioning the existence of a bilingual advantage.

Finally, we were interested in examining whether there would be interactive effects of each skill type and we explored the possibility that being both musically trained and bilingual might confer additive benefits compared to having only one skill type. Given that musical training and bilingualism display some separable effects on cognition, it is possible these effects may interact. One option is that the benefits from each domain may combine to produce an additive effect. Alternatively, each domain may produce a maximal effect, such that no further benefits are possible. Evidence on word learning supports the latter – music and speaking a tonal language have independent and non-cumulative effects (Cooper & Wang, 2012).

## Methods

### Participants

Participants (*n* = 153) were recruited through advertisements posted at Toronto universities and through professional contacts and were paid $30 for their time. Participant characteristics are presented in Table 1. The sample included 98 women and 55 men (age range = 18–31 years; mean = 22.0, standard deviation [SD] = 2.9). None reported being colorblind. Participants were carefully screened, and those who met the inclusion criteria were grouped according to their expertise into monolingual musicians, monolingual non-musicians, bilingual musicians, and bilingual non-musicians.

**Table 1:** Participant characteristics with means (SD) for each grouping variable

	Musician		Non-musician	
	Monolingual	Bilingual	Monolingual	Bilingual
n	45	36	36	36
Age (years)	21.5 (3.1)	22.5 (3.2)	22.6 (2.6)	21.5 (2.3)
SES (mother’s education)^a^	3.4 (1.1)	3.4 (1.4)	2.7 (1.3)	3.4 (1.3)
Father’s education^a^	3.2 (1.4)	3.6 (1.6)	2.5 (1.9)	3.9 (1.2)
Parents’ income^b^	6.1 (2.0)	5.4 (2.4)	6.5 (2.3)	5.4 (2.7)
K-BIT-2 vocabulary (raw)	51.0 (3.4)	49.2 (3.6)	49.7 (4.4)	45.0 (4.4)
K-BIT-2 matrices (standardized)	103 (22.3)	105 (14.7)	104 (15.2)	101 (12.5)

^a^Education was in the range of 0–5 (0 = high school not completed; 1 = high school diploma; 2 = some college; 3 = college diploma; 4 = Bachelor’s degree; 5 = graduate or professional degree)

^b^Parents’ income was in the range of 1–11 (1 ≤ $14,900; 2 = $15,000–29,000; 3 = $30,000–44,900; 4 = $45,000–59,900; 5 = $60,000–79,900; 6 = $80,000–99,900; 7 = $100,000–129,000; 8 = $130,000–159,000; 9 = $160,000–179,000; 10 = $180,000–199,9000; 11≥ $200,000)

*SES* socioeconomic status

Sample size was determined based on an a priori power analysis and was designed to have 80% power to detect both predicted main effects and the predicted interaction, based on effect sizes of studies reported in prior literature in the target populations of interest. G*Power (Faul, Erdfelder, Buchner, & Lang, 2009) was used to determine sample size. According to the software, a total sample size of *n* = 128 would be required (i.e. *n* = 32, with four groups) to obtain a medium to large effect size of Cohen’s *f* = 0.25 (Cohen, 1988), based on then-published literature, for an analysis of variance (ANOVA; fixed effects, special, main effects and interactions, α-level: 0.05, power [1-β error probability]: 0.80). In this study, we exceeded the necessary sample size. However, a more conservative effect size might have been preferable given null evidence that has emerged in the bilingualism literature since we ran the study.

Bilinguals were fluent in English plus at least one other language (Arabic, Armenian, Bulgarian, Cantonese, Farsi, French, German, Ghanian, Greek, Gujarati, Hebrew, Hindi, Italian, Indonesian, Japanese, Kachi, Korean, Mandarin, Portugese, Punjabi, Romanian, Russian, Serbian, Sinhalese, Spanish, Tibetan, Turkmen, Twi, Urdu, Vietnamese, Yoruba, Zulu). Bilinguals were asked to describe themselves on level of bilingualism on a 5-point scale (i.e. 1 = speak only one language; 2 = weak bilingual; 3 = unbalanced bilingual; 4 = practical bilingual; and 5 = fluent bilingual). All bilinguals were able to speak and understand both languages; and 93% were either practical bilinguals (i.e. can carry out conversation fluently but do not use second language daily) or fluent bilinguals (i.e. able to converse fluently and actively use two languages every day). Crucial factors in defining bilingualism are fluency and proficiency (Luk & Bialystok, 2013). On both of these factors, the level of bilingualism was high and matched previous studies (Bialystok, 2009; Paap & Greenberg, 2013). Individuals were considered to be monolingual if they were fluent only in the English language, with little or no training in a second language. Other language factors that were previously shown to have no effect (such as multilingualism or age of onset; Pelham & Abrams, 2014) were not measured.

Musicians included individuals who had at least eight years of experience playing and performing music, began training at age seven years on average, and regularly practiced music (an average of 6–9 h a week). Musicians had, on average, 12 years of formal musical training, using the Royal Conservatory of Music curriculum or similar, and length of training was in the range of 7–22 years. Moreover, 90% had music theory training, 83% had ear training, and on average musicians rated themselves 3.25 or having “good” sight-reading ability on a 5-point scale where 1 = “beginner” and 5 = “expert.” The majority of musicians in the sample (96%) began their training before the age of 12 years. Musicians consisted of instrumentalists (88%) who played at least 1 of 17 instruments (bass, cello, clarinet, drums, flute, guitar, keyboard, organ, piano, saxophone, shamisen, steel drum, trombone, trumpet, ukulele, viola, violin) and vocalists (12%). Non-musicians included individuals with little (< 2 years) or no exposure to musical training; none currently practiced or performed music.

### Measures

#### Background questionnaires

Participants completed a detailed self-report questionnaire about their music, language, and demographic background before completing the experimental tasks. The music background questionnaire included questions regarding the age at which participants began taking music lessons, the duration of training, the frequency and duration at which they practiced music on a weekly basis, and the level of sight-reading, ear training, and music theory achieved. The language background questionnaire included questions regarding what languages the participant could speak and understand, the frequency of language use, and the context and proportion of use of the languages spoken (i.e. percentage of time spent talking, listening, and reading and the language used at home and work/school). Finally, demographic questions inquired about the level of education completed by the participant and the participant’s parents, the participant’s daily use of computer or video games, involvement in sports and other physical activities, and general health.

#### Non-verbal intelligence and vocabulary

Vocabulary and non-verbal intelligence were assessed using the Kaufman Brief Intelligence Test-2 (K-BIT-2; Kaufman & Kaufman, 2004). The Matrices subtest of the K-BIT-2 is a standardized measure of non-verbal fluid intelligence. In this task, a series of abstract images were presented and participants were required to complete visual analogies by indicating the relationship between images. The Verbal Knowledge subtest of the K-BIT-2 was used to examine receptive vocabulary. In this task, participants were presented with a word or phrase and they were required to choose a picture that corresponded to that word or phrase. This task required no reading or spelling on the part of the participant. Both the Matrices and Verbal Knowledge subtests were administered and scored according to the K-BIT-2 manual and standardized Matrices scores were obtained for participants. We did not administer the Riddles subtest, so Verbal Knowledge scores are raw, not standardized.

#### Working memory

The digit span (Wechsler, 1981), reading span (Unsworth, Heitz, Schrock, & Engle, 2005), and operation span (Unsworth et al., 2005) tasks were used to measure working memory. The digit span task is a subtest of the Wechsler Adult Intelligence Scale (WAIS) and consists of digit span forward and digit span backward. We were interested in performance on digit span backward, which is considered a measure of executive or complex working memory (George & Coch, 2011). In the digit span backward, participants were verbally presented with a series of numbers at a rate of one digit per second by an examiner and they were required to immediately repeat them back in the reverse order. If the participant correctly recalled the sequence, the number of digits presented increased by one in the following sequence. This procedure was repeated until participants recalled a set of two-digit sequences inaccurately. The total number of digit sequences correctly recalled by participants was measured and the task took approximately 5 min to complete.

In the automated reading span task, participants were required to determine whether the sentences they read made sense while trying to remember a string of unrelated letters. For example, participants were first presented with a sentence, such as “the young pencil closed his eyes,” and they were required to indicate whether the sentence made sense by answering true or false. Next, letters were individually presented on the screen and participants were required to remember the letters and the sequence in which they appeared. The presentation of sentence and letters alternated until a recall screen was presented, where participants were required to recall the letter sequences. If a participant forgot a letter, they had the option to mark the position of missing letters by pressing the “blank” button provided on the screen. There were 10–15 words in each sentence. The number of letters to be recalled varied from set to set, in the range of 3–7 letters, and 81 sentence problems were presented. Practice sessions were provided, including 15 sentence problems as well as letter recall, and participants were given feedback. The total number of letters correctly recalled in the correct position was measured, and the task took approximately 20–25 min to complete.

In the automated operation span task, participants were required to remember a string of letters while solving simple math questions. For example, participants were required to compute a simple math question (e.g. 2*1 + 3) in mind as quickly as possible. On the following screen, a number was presented. If the number on the screen was the correct answer to the math question, participants clicked the box identified as “true,” and if it was the wrong answer, participants clicked on the box identified as “false.” Then a letter appeared on the screen (lasting 800 ms), and participants were required to remember the letter. At the end of each set of math questions and letters, a recall screen appeared where participants were presented with a matrix of letters where they were required to recall the letters they were presented with and in their correct order. If a participant could not recall a letter within the sequence, there was an option to mark the position of missing letters by pressing the “blank” button provided on the screen. The number of letters to be recalled and math problems to be solved varied from set to set, in the range of 3–7 letters or math problems for a total of 75 math problems and 75 letters. Practice sessions for the math and letter portions of the task were also provided beforehand consisting of 15 simple math questions and feedback was provided on the practice trials. The scoring procedure and total time to complete the task was the same as that of the automated reading span task.

#### Inhibitory control

The Stroop (Stroop, 1935) and flanker (Eriksen & Eriksen, 1974) tasks were used to measure interference control. The Stroop task is used to examine how well individuals can suppress interfering information or stimuli in order to select the appropriate response. The version of the Stroop task we used (Cepeda, Blackwell, & Munakata, 2013) involved three sets of stimuli: (1) non-color words (i.e. words that do not refer to colors) printed in red, orange, yellow, green, blue, or purple (e.g. the word “advice” printed in blue ink). These were considered to be congruent since non-color words were not expected to interfere with naming the ink color; (2) color words (i.e. words that refer to colors) printed in ink that differed from the color that the word described (e.g. the word “purple” printed in red ink). These were considered incongruent, because there is greater interference when trying to identify the ink color and not read the word; and (3) asterisks printed in color (e.g. ***** printed in green ink). This baseline set of trials was expected to produce the fastest reaction times (RTs) because there was no interference or conflict, such as word reading, when naming the ink color of the stimuli. Stimuli were presented to participants on sheets of paper and they were required to read the ink colors out loud. The examiner recorded how long (in seconds) it took participants to complete each page using a stopwatch. Each set of stimuli contained 60 items. Initially, participants were presented with practice trials requiring them to name the ink colors of 12 incongruent word stimuli (color words printed in different color ink; no participants reported colorblindness). The final interference score was computed by subtracting the time it took to read the non-color words from the time it took to read the color words. The task took approximately 5 min to complete.

In a modified version of the flanker task (Bialystok et al., 2017; Bunge, Dudukovic, Thomason, Vaidya, & Gabrieli, 2002), participants were asked to respond to the direction of a red target chevron either on its own or among flanker stimuli. If the target arrow pointed to the left, participants had to press the far left mouse button with their left index finger on a mouse located to the left of a keyboard. If the target arrow pointed to the right, participants had to press the far right mouse button using their right index finger on another mouse located to the right of the keyboard. There were three parts to the task. The first part consisted of the null condition, where participants were presented with a red chevron and were required to indicate its’ direction using a mouse button (e.g. < or >). The second part consisted of neutral conditions, where participants were presented with a red chevron that was flanked by black diamonds (e.g. <> <> < <> <>), and participants were required to indicate the direction of the chevron despite the presence of black diamonds. The null and neutral conditions were non-experimental in nature since there were either no flankers present or the presence of flankers did not interfere with the participant’s response. Both conditions contained six practice trials, which included feedback on trials, and 24 experimental trials. In the third part of the task, both congruent and incongruent conditions were mixed together in one block and consisted of 12 practice trials and 48 experimental trials. In the congruent condition, a red chevron was flanked by four black chevrons pointing in the same direction as the target (e.g. < < < < <); in the incongruent condition, a red chevron was flanked by four black chevrons pointing in the opposite direction of the target (e.g. < < > < <). In this task, participants’ reaction time was measured. The fixation duration was 250 ms and the stimulus duration was 2000 ms. Once the stimulus disappeared from the screen, the trial automatically progressed regardless of whether a response was made. The task took approximately 10 min to complete.

The stop signal task (Blackwell, Chatham, Wiseheart, & Munakata, 2014) was used to measure response inhibition. First, in a reaction time block consisting of 16 trials, participants were required to press the letter “f” on the keyboard when they saw a blue circle on the screen or the letter “j” when they saw a red circle, as quickly and accurately as possible. No cues were provided and the mean reaction time was used as a baseline to compare performance on the stop signal trials. Next, participants performed a practice stop signal block consisting of 16 trials, where they were presented with go and no-go trials. On the no-go trials, participants were required to inhibit their response (i.e. not press “f” or “j” in response to the circle on the screen) when they heard a stop signal (i.e. the verbal prompt “stop”). This block allowed participants to become better acquainted with the stop signal portion of the task and allowed for calibration of the stop signal delay.

In the experimental part of the task, consisting of three blocks of 48 trials, participants heard a stop signal (i.e. the verbal prompt “stop”) on 25% of trials, which required them to inhibit their response to the go task. On another 25% of trials, a go signal (i.e. the verbal prompt “go”) was presented and on 50% of trials no prompt was provided (identified as no signal). During all stop signal blocks, the stop signal delay was iteratively adjusted up or down by 50-ms intervals, depending on whether the participant successfully stopped their response, with the goal of producing 50% successfully stopped trials in response to the stop signal. When the participant’s RT to the primary task was more than 2.3 SDs longer than the mean initial choice RT, a verbal prompt (“faster!”) reminded participants to respond as quickly as possible.

Stop signal reaction time (SSRT) was used as a measure of individual response inhibition. SSRT is an estimate of the time it takes for a stop process to finish once it is initiated by a stop signal. The finishing time of the stop process was estimated by taking the *i*th percentile of the go trial distribution, where *i* corresponded to the percentage of successfully stopped trials in the stop signal condition (Ridderinkhof, Band, & Logan, 1999). The average stop signal delay was subtracted from the estimated finishing time of the stop process in order to determine the stop signal reaction time. A smaller SSRT indicated faster inhibition when responding to the primary task. The task took approximately 10 min to complete.

### Procedure

The working memory and inhibitory control paradigms were a part of a larger battery consisting of 11 tasks that measured various executive constructs. The task battery took approximately 2 h to complete and the tasks were presented to participants in a fixed order, beginning with informed consent, followed by the background questionnaires, the cognitive paradigms, and lastly, debriefing of participants. Cognitive paradigms were the digit span, reading span, stop signal, and Stroop tasks, plus those reported in an earlier publication^1^ (Moradzadeh, Blumenthal, & Wiseheart, 2014). The computer-based tasks were presented on a Microsoft Windows XP computer and displayed on a 15-inch (1280 × 1024 pixels) monitor.

Two extra tasks were added to the battery to improve the initial choice of tasks. First, the flanker task was added to the battery one-third of the way through initial data collection and data were collected for 95 participants on this task (Appendix 1). After testing had been completed, we realized that one of the tasks (the reading span) may have unfairly disadvantaged the bilingual group due to the task’s emphasis on English language ability. Hence, a non-verbal task (the operation span) was added to the battery to clarify any issues that bilinguals may have had on the reading span task and assess whether data from this task corroborated the initial findings. The initial participants were re-contacted one year after commencing data collection to see if they would be willing to participate in an additional task. Of the 153 participants in the original study, 54 participants could be reached and agreed to partake in the follow-up session. Of this subset, all participants had partaken in the first testing session and demographic variables for this group were consistent with the initial 153 participants.

## Results

### Analyses

Between-subjects 2 × 2 (musician status [musician, non-musician] × language status [bilingual, monolingual]) ANOVAs and Bayesian analyses were conducted for each measure. Data analyses for all tasks were performed using more stringent classification criteria for bilingualism and musician status (Appendix 2 and 3). These secondary analyses produced results consistent with the ones reported in the primary text.

To account for using multiple measures, we used the step-wise Bonferroni-Holm procedure.^2^ Based on guidelines for multiplicity (Cribbie, 2017) the alpha level was adjusted across all the tests for each construct but not across constructs, since we were interested in how our independent variables (musical training or bilingualism) affected each construct separately. Adjusted significance values at an alpha level of 0.05 for three measures per construct were *p*_*adjusted*_ = 0.017*, p*_*adjusted*_ = 0.025*,* and *p*_*adjusted*_ = 0.05, in order of the size of obtained effects. Results remained significant after accounting for multiplicity.

### Background variables

There were no significant differences between groups on age, *F*(3, 148) = 1.63, *p* = 0.185, or SES (as measured by mother’s education level), *F*(3, 149) = 2.24, *p* = 0.086 (Table 1). Bayesian analyses also showed no notable effect across age for music, language, or an interaction, BF_Inclusion_ = 0.15, BF_Inclusion_ = 0.15, BF_Inclusion_ = 0.16, respectively, nor for SES, BF_Inclusion_ = 0.43, BF_Inclusion_ = 0.34, BF_Inclusion_ = 0.25. Non-verbal IQ did not differ between groups, *F*(3, 148) = 0.26, *p* = 0.857, similarly for Bayesian analyses for music, language, or an interaction, BF_Inclusion_ = 0.35, BF_Inclusion_ = 0.22, BF_Inclusion_ = 0.08, respectively. Vocabulary score was significantly higher in musicians compared to non-musicians, *t*(70) = 2.88, *p* < 0.005, *d* = 0.68, BF_Inclusion_ = 5.64, and higher among monolinguals compared to bilinguals, *t*(70) = 3.47, *p* < 0.001, *d* = 0.82, BF_Inclusion_ = 25.05, with moderate to strong differences supported by Bayesian analyses.

### Working memory

Descriptive statistics for working memory measures are presented in Table 2. Due to the verbal nature of the reading span task, we covaried reading span scores with receptive vocabulary (measured using the K-BIT-2). The covariate, receptive vocabulary, was significantly correlated with reading span score, *F*(1, 66) = 5.39, *p* = 0.023, η^2^ = 0.006, BF_Inclusion_ = 3.44, and removed 15% of error variance (calculations based on Sabers & Franklin Jr, 1986). Accounting for the correlation helped isolate the effect of the independent variables (Owen & Froman, 1998) and thus answers issues raised on the suitability of verbal tasks for bilinguals. While the covariate had some overlap with the independent variable, the effects of the grouping variable were meaningful beyond the overlap which justifies use of the covariate (Zinbarg, Suzuki, Uliaszek, & Lewis, 2010).

**Table 2:** Mean correct items (SD) on working memory measures by participant group

		Digit span backward	Reading span	Operation span
Musician	Monolingual	8.74 (2.24)	39.6 (16.9)	52.7 (17.7)
	Bilingual	8.11 (2.40)	37.4 (19.8)	37.0 (14.3)
Non-musician	Monolingual	7.72 (2.93)	24.8 (14.3)	26.9 (18.1)
	Bilingual	7.15 (2.26)	22.3 (10.7)	45.6 (23.6)

**Fig. 1:**
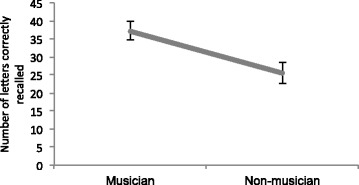
Musician status in the reading span task. Musicians recalled more letters, that is, had a larger span, than non-musicians. *Error bars* represent SEM

The reading span task showed a main effect of musician status, *F*(1, 66) = 8.74, *p* = 0.004, with a small to medium effect size, η^2^ = 0.098, and strong evidence from Bayesian analyses, BF_Inclusion_ = 18.27. Musicians correctly recalled more items (mean = 37.2, SEM = 2.6) than non-musicians (mean = 25.5, SEM = 2.9), after controlling for the effects of receptive vocabulary (Fig. 1). Analyses of covariance controlling for receptive vocabulary found neither a significant main effect of language status nor a significant musician by language status interaction.

**Fig. 2:**
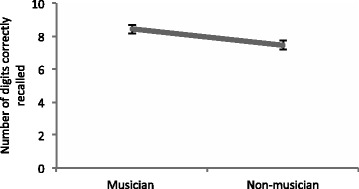
Musician status in the digit span backward task. Musicians recalled more digits, that is, had a larger working memory span than non-musicians. *Error bars* represent SEM

The digit span backward task showed a main effect of musician status, *F*(1, 148) = 6.07, *p* = 0.015, and a small effect size, η^2^ = 0.04. Musicians correctly recalled more items (mean = 8.43 items, SEM = 0.274) than non-musicians (mean = 7.44 items, SEM = 0.294; Fig. 2). No other effects reached significance. Bayesian analyses supported an inconclusive but possible music effect, BF_Inclusion_ = 2.31, and inconclusive to no effects for language or an interaction.

**Fig. 3:**
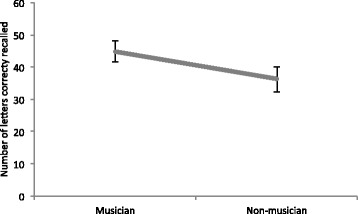
Musician status in the operation span task. Musicians recalled more letters, that is, had a larger working memory span than non-musicians. *Error bars* represent SEM

The operation span task showed a main effect of musician status, *F*(1, 50) = 4.62, *p* = 0.018, with a medium to large effect size, η^2^ = 0.11, and strong evidence from Bayesian analyses, BF_Inclusion_ = 11.93. Musicians correctly recalled more items (mean = 44.8, SEM = 3.25) than non-musicians (mean = 36.2 items, SEM = 4.00; Fig. 3). No main effect of language was demonstrated. The analysis showed an interaction between musician and language status, *F*(1, 50) = 5.44, *p* = 0.024, with a medium to large effect size η^2^ = 0.16, and strong evidence from Bayesian analyses, BF_Inclusion_ = 17.85. A follow up *t-*test demonstrated higher accuracy in monolingual musicians (mean = 52.7, SEM = 4.14) compared to monolingual non-musicians (mean = 26.9, SEM = 4.82) on this task, *t*(30) = 3.70, *p* = 0.001. However, no significant difference was found on this task between bilingual musicians and non-musicians (*p* > 0.940). Bayesian analyses also found moderate evidence for a language effect, BF_Inclusion_ = 4.04.

### Inhibitory control

Descriptive statistics for inhibitory control measures are presented in Table 3.

**Table 3:** Mean reaction times (SD) on inhibitory control measures by participant group

		Stroop^a^ (s)	Stop signal SSRT (ms)	Flanker^a^ (ms)
Musician	Monolingual	12.3 (6.18)	281 (61.2)	54.0 (24.0)
	Bilingual	14.3 (8.28)	271 (69.7)	60.8 (36.5)
Non-musician	Monolingual	15.5 (6.03)	284 (54.8)	69.3 (46.2)
	Bilingual	15.4 (7.25)	292 (62.3)	61.5 (38.7)

^a^Difference scores on the Stroop and Flanker tasks were calculated by subtracting congruent from incongruent conditions

**Fig. 4:**
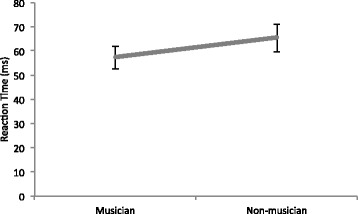
Musician status in the flanker task. Musicians outperformed non-musicians on null, neutral, congruent, and incongruent conditions. *Error bars* represent SEM

On the flanker task, a difference score was calculated by subtracting the mean reaction times for congruent trials from incongruent trials. Results showed a main effect of musician status, *F*(1, 92) = 8.18, *p* = 0.005, with a small effect size, η^2^ = 0.012, with faster response times (i.e. smaller difference scores) among musicians (mean = 57.4 ms, SEM = 4.78) than non-musicians (mean = 65.4 ms, SEM = 5.76; Fig. 4). Bayesian analyses revealed inconclusive congruency by music interaction, BF_Inclusion_ = 0.81, demonstrating that even though the *p* value is significant, the effect magnitude is not meaningful to interpret. No other effects reached significance.

A secondary analysis was run to compare music and language groups on baseline conditions of the flanker task (average of null and neutral) and mixed conditions (average of congruent and incongruent). Musician groups were significantly faster than non-musician groups on baseline conditions, *F*(1, 91) = 7.06, *p* = 0.009, η^2^ = 0.07, and on mixed conditions, *F*(1, 91) = 8.02, *p* = 0.006, η^2^ = 0.08. No differences were found between language groups on any of the conditions (*ps* > 0.406). Independent samples *t*-tests examining performance of musicians and non-musicians on the flanker task revealed significant differences in RT on all conditions, including null (*t*(94) = − 3.04, *p* = 0.003), neutral (*t*(94) = − 2.25, *p* = 0.027), congruent (*t*(94) = − 2.57, *p* = 0.012), and incongruent (*t*(94) = − 3.10, *p* = 0.003) trials, suggesting a possible processing speed advantage on musicians across on response times for all types of trials, including null, neutral, congruent, and incongruent conditions. However, since a similar benefit was not seen on the other measures, a speed of processing benefit remains inconclusive.

A third analysis was conducted to compare our findings to previous findings that used an identical version of the task (Bialystok et al., 2017). A proportion score was calculated by subtracting baseline conditions from mixed conditions and dividing by the baseline conditions. No difference was found in either music or language groups on the proportion score (*p*s > 0.35).

Difference scores on the Stroop task were calculated by subtracting congruent from incongruent conditions. The Stroop task showed a trend towards significance for musician status, *F*(1, 147) = 3.56, *p* = 0.061, with a small effect size, η^2^ = 0.023. Bayesian analyses revealed an inconclusive music effect, BF_Inclusion_ = 0.77, so the marginal *p* value should not be interpreted. No other effects reached significance.

The stop signal task demonstrated no significant differences between musicians and non-musicians in their performance on no signal trial SSRTs in the stop signal task (*p* > 0.22). We considered the possibility that some musicians may not have been as proficient in an orchestral or group musical environment where response inhibition is necessary. Thus, in order to analyze differences on response inhibition in the relevant musician group, the performance of musicians who had specifically indicated that they were a part of an orchestra or band was compared to those without musical training on the stop signal task. Results of this follow-up analysis also did not indicate any musician benefits on SSRT, *p* > 0.76. No other effects reached significance, all *p*s > 0.90, all BF_Inclusions_ < 0.33.

## Discussion

The present study investigated the association between musical training and bilingualism, both specialized forms of auditory experience, in two areas of executive function: working memory and inhibitory control. Existing evidence has indicated the potential for a cognitive advantage from both skills but has been inconclusive on whether effects are specific to a certain skill type or cognitive ability. Our results demonstrate that musical training is associated with an advantage on working memory but not on inhibitory control. The findings failed to show a bilingual advantage across working memory and inhibitory control tasks, nor a combined benefit of being both bilingual and musically trained. The current findings suggest that learning a skill can influence cognition but that effects are specific to some skills and some cognitive abilities.

Our findings help clarify uncertainty in the existing literature on the specificity of effects. First, we employed multiple tasks for each construct, enabling clearer insights into the target constructs beyond task-specific effects. Examining convergent validity among tasks measuring the same constructs resolved ambiguities in previous findings by confirming whether robust effects can be found for musical training and bilingualism. Second, we directly compared two skill types to show that effects can be uniquely attributed to some skills over others. Our results add to increasing evidence on the lack of replication of a bilingual advantage and limited effects of a musician advantage.

Findings of a musician effect on working memory across three tasks (digit span backward, reading span, and operation span) are consistent with previous studies and extend existing evidence to both simple and complex tasks. Findings demonstrated no musical training or bilingualism benefits on interference control across three tasks (flanker, Stroop, and stop signal). The absence of a bilingual advantage in inhibitory control is not surprising given the recent increase in reporting an inability to replicate original findings on a benefit (Paap et al., 2015). A common limitation in the literature has been the use of a single task or paradigm, which limits the generalizability of the findings by measuring only one form of the target construct. Our tasks used varying stimuli suggesting that effects go beyond measurement error or task selection.

It was not clear, a priori, whether speaking a second language and musical training should be associated with similar patterns of cognitive functioning. On one hand, music and language involve overlapping processing systems and have demonstrated transfer from one domain to the other (Asaridou & McQueen, 2013; Besson et al., 2011; Moreno, 2009; Patel, 2011, 2014). On the other, they demonstrate notable differences due to the varying functional demands of each domain (Hutka et al., 2013). Our findings help address the debate on the similarity between music and language and support a distinction between music and language since we did not observe similar patterns of performance. While, music effects were demonstrated across cognitive measures of working memory, bilingual effects were not. The current study demonstrates the unique vantage point of comparing domain-general enhancement from one training form compared to another.

### No evidence for a bilingual advantage on cognition

In line with emerging evidence suggesting no bilingual advantage on cognitive tasks, our study failed to demonstrate associations between being bilingual and improved performance on working memory or inhibitory control. Our findings address the main two proposed reasons for null effects in the bilingual literature: use of single tasks and a “peak advantage” of bilingual young adults (Bialystok, Martin, & Viswanathan, 2005; Paap & Greenberg, 2013). Both reasons fail to explain the lack of a bilingual advantage in the current study, since multiple tasks were used and young adults did show a benefit on musical training, adding further skepticism to the notion that a general bilingual advantage exists beyond task-specific or sample-specific constraints.

Beyond task effects, another debate is that the bilingual advantage may be restricted to certain individuals. A few previous studies have found a larger bilingual advantage in older than in younger adult participants, but most did not (c.f. Abutalebi, Canini, Della Rosa, Green, & Weekes, 2015; Bialystok et al., 2008; Bialystok, Craik, Klein, & Viswanathan, 2004; Faroqi-Shah, Sampson, Pranger, & Baughman, 2016; Gold et al., 2013, Exp. 2). Notably, a bilingual effect in older adults has not been consistently replicated, while benefits of musical training have been found across age groups.

One explanation for null effects in young adult bilinguals is that young adults are already at maximal executive function performance (Bialystok et al., 2005). The “functional ceiling” was proposed due to the number of opportunities available for individuals to reach peak cognitive performance, including daily demands such as inhibiting distractions and unsuitable responses (Bialystok, Craik, & Luk, 2012). However, there is no research demonstrating that the hypothesized asymptotic limit exists for executive function performance, and a direct test of the ceiling hypothesis found within experiment improvement (Paap, Johnson, & Sawi, 2014; Paap et al., 2014, May), thus challenging the hypothesis. Our demonstrated musical training benefits, even in young adults, support similar musician but not bilingual benefits in our previous study with undergraduates (Moradzadeh et al., 2014) and suggests that an age limit explanation does not hold.

It has been argued that the task selection may hide a bilingual advantage (Bialystok, 2009). One factor is complexity of the task, with the bilingual advantage being strongest with complex tasks requiring substantial amounts of executive function (Bialystok, 2009, 2011). Simple working memory and short-term memory tasks showed no difference in bilinguals and monolinguals (Bialystok & Feng, 2009). However, others found the opposite, where a bilingual advantage was seen on a simple but not complex version of the Simon task (Salvatierra & Rosselli, 2010). Granted, these are different tasks, but the set of findings still challenges the hypothesis of a general benefit in bilinguals. Despite using simple and complex forms, no bilingual benefits were found in our study. In comparison, musical training demonstrated improvements on both simple and complex working memory measures, consistent with previous data indicating the same (Lee et al., 2007). Another proposition is that the bilingual advantage can only be observed under certain experimental conditions—for example, on high-monitoring conditions in the flanker task, or in baseline compared to mixed conditions (Bialystok et al., 2017; Costa, Hernández, Costa-Faidella, & Sebastián-Gallés, 2009). However, we used the same tasks and analysis procedure as previous investigations and could not find a bilingualism advantage.

Another factor is verbal content of tasks, which could even reverse a bilingual advantage (Bialystok, 2009; Bialystok, Poarch, Luo, & Craik, 2014). Correlations of vocabulary with reading span scores in our study echo concerns that bilinguals may be disadvantaged on verbal measures. However, even after following recommendations to covary vocabulary level (Barac, Bialystok, Castro, & Sanchez, 2014), no change in results was seen, which suggests that possible verbal deficits in bilinguals did not affect performance on the reading span task. Further, we did not find an effect on complex non-verbal tasks—for both the operation span task, a non-verbal measure of working memory, or the digit span backward, an auditory measure. Our null effects match those of Ratiu and Azuma (2015) who also did not find a bilingual benefit when using complex non-verbal tasks. Even where an effect was previously found on a non-verbal task, it was not consistent. For example, bilinguals performed significantly better on a backward version of the Corsi blocks task, but not on the forward version, or on the self-ordered pointing task, a complex non-verbal measure (Bialystok et al., 2008). Overall, beyond task-specific effects (verbal content or complexity), for a coherent benefit to occur it would need to be demonstrated across measures of a construct.

While previously proposed conditions for a bilingual advantage were not supported by our investigation, it is possible that our study may have missed a factor that is required for a bilingual advantage. We concur that a bilingual advantage may be generated under very specific conditions, but the very large number of suggested conditions and requirements shows that any effect is the exception rather than the norm and may be a statistical artifact. Nevertheless, learning a language has intrinsic benefits regardless of whether cognitive benefits exist.

### Methodological considerations

Several caveats should be noted in relation to the current findings. First, the correlational design of the present study prevents us from knowing whether musicians had pre-existing differences from non-musicians before undergoing musical training. Although multiple tasks per measure were used, due to the unbalanced sample sizes across tasks we could not run a factor analysis or multivariate ANOVA. An analysis would have been ideal to account for individual variability between groups and any co-linearity between variables, but would have produced a considerable loss in power as it would need to use the smallest possible sample size across task. However, a multivariate analysis was conducted by combining data across tasks to remove individual differences and found no change in the pattern of results. Future research could address this with larger sample sizes and multivariate analyses.

The current findings are limited to the classifications of musical training and bilingualism used. Musicians were defined as individuals who had formal musical training of at least eight years (the final sample had an average of 12 years of training). The amount and type of training matches previous studies (Fujioka et al., 2004; Lee et al., 2007; Schellenberg, 2011), with a minimum number of years being important since extra-musical effects are linked to the duration of training (Wan & Schlaug, 2010). Since effects of musical training on cognition were found to not vary for instrumental and vocal training (Bialystok & DePape, 2009), we did not distinguish between these. Similarly, our study based the definition of bilinguals (proficiency and frequency of usage) on empirically supported components of language use (Luk & Bialystok, 2013). Other language factors that were previously shown to have no effect (such as multilingualism or age of onset; Pelham & Abrams, 2014; Vega-Mendoza, West, Sorace, & Bak, 2015) were not measured.

The classification criteria we used for musicians and bilinguals were selected to make both groups qualitatively similar. Rather than quantify both groups on the same measurements of experience for each skill, we chose to use the strongest available definitions in the independent bilingualism and music literature to classify each, as this provides reliable definitions that also connect to previous findings. Although the definitions used mean that the measure of experience for skill form is not directly matched, they enabled us to measure individuals who had high levels of ability on their respective skills forms (either language or musical training), rather than being comparable only on quantitative information, but different in their level of ability. Hence, we do not think that similar quantitative information is comparable. For example, four years of musical training is qualitatively different to four years of bilingual experience. Comparable quantitative details on how both types of experience relate to other participant characteristics are presented in Table 1. Future research can investigate how within-group variability in training influences results.

## Conclusions

Individuals are exposed to a number of experiences and factors that can improve executive function. Musical training and bilingualism are two such examples. The current study tested the uniqueness of training effects from each of these skill domains. Our findings suggest that long-term musical training is associated with advantages in working memory ability, with benefits found across several measures indicating a consistent benefit. In contrast, bilingualism was not associated with benefits in these domains relative to being monolingual, nor was there an effect of musical training on interference control, or an interactive effect of both skills. Our results on the lack of a bilingual advantage should be noted, especially given that the same sample had the capacity to demonstrate an advantage from other activity. Examining connectivity across the two training domains provided a distinctive comparative insight to elucidate the robustness of any effects.

## Appendix 1

**Table 4:** Breakdown of sample size for the operation span and flanker tasks

Participant groups	Operation span (*n* = 54)	Flanker (*n* = 95)
Monolingual musicians	19	33
Monolingual non-musicians	14	20
Bilingual musicians	13	22
Bilingual non-musicians	8	20

## Appendix 2

### Musical practice re-analysis

Given that a significant difference was found between monolingual and bilingual musicians on current weekly hours of music practice, it was important to account for this variable. However, this variable could not be included as a covariate in the analysis since this would mean that non-musicians would have zero hours of current practice, which would result in covariate data for only half of the sample. In order to effectively account for this covariate, participants were split into three groups, consisting of non-musicians, low-practicing musicians, and high-practicing musicians, to determine whether hours of current musical practice had a significant effect. In order to classify musicians as high- or low-practicing, the median number of hours of weekly music practice was used to split the musician sample into two equal sized groups. These follow-up analyses were identical to the original analyses performed, except that the musician group was divided into three separate levels (high-practicing, low-practicing, and non-musician) rather than two levels (musician, non-musician).

### Analyses

#### Working memory

**Digit span.** When utilizing this particular grouping method, results of a 2 × 3 (language status [bilingual, monolingual] × musician status [high-practicing musician, low-practicing musician, non-musician]) between-subjects ANOVA for the digit span forward task demonstrated a main effect of language, *F*(5, 146) = 11.27, *p* = 0.001, η^2^ = 0.072, which is a small to medium effect size, with monolinguals outperforming bilinguals, and a marginal main effect of musician status, *F*(5, 146) = 2.73, *p* = 0.069, η^2^ = 0.036, which is a small effect size, with musicians outperforming non-musicians. There was no significant interaction between musician and language status. Post-hoc comparisons using the Tukey HSD test indicated a significant difference between high-practicing musicians and non-musicians (*p* = 0.02) on mean accuracy scores. However, mean score differences between high- and low-practicing musicians were non-significant (*p* = 0.658), as was the difference between low-practicing musicians and non-musicians (*p* = 0.195). Results of the digit span backward demonstrated a marginal main effect of musician status, *F*(5, 146) = 2.64, *p* = 0.075, η^2^ = 0.035, with musicians outperforming non-musicians. Post-hoc Tukey HSD comparisons of musician status demonstrated a significant mean difference between high-practicing musicians and non-musicians (*p* = 0.041), while differences between high- and low-practicing musicians (*p* = 0.807) and that of low-practicing musicians and non-musicians (*p* = 0.192) were non-significant. Finally, there was neither an interaction between musician and language status, nor a main effect of language status.

**Reading span.** A 2 × 3 (language status [bilingual, monolingual] × musician status [high-practicing musician, low-practicing musician, non-musician]) between-subjects ANOVA was performed. Results demonstrated a main effect of musician status, *F*(5, 143) = 4.36, *p* = 0.015, η^2^ = 0.057, with musicians outperforming non-musicians, and a main effect of language status, *F*(5, 143) = 5.22, *p* = 0.024, η^2^ = 0.035, with monolinguals outperforming bilinguals. A significant interaction between music and language status was not demonstrated. Post-hoc comparisons using the Tukey HSD test indicated a significant difference between high-practicing musicians and non-musicians (*p* = 0.001). However, the performance of high-practicing musicians was not significantly different from that of low-practicing musicians (*p* = 0.112), nor was the difference in performance between low-practicing musicians and non-musicians (*p* = 0.324).

**Operation span.** A 2 × 3 (language status [bilingual, monolingual] × musician status [high-practicing musician, low-practicing musician, non-musician]) between-subjects ANOVA was conducted. Results demonstrated a main effect of musician status, *F*(3, 48) = 3.62, *p* = 0.034, η^2^ = 0.13, with musicians outperforming non-musicians. An interaction between music and language status was no longer demonstrated once level of practice was factored in. Post-hoc comparisons using the Tukey HSD test indicated that there was a significant difference between high-practicing musicians and non-musicians (*p* = 0.006) on mean accuracy scores. However, mean score differences between high- and low-practicing musicians were non-significant (*p* = 0.321), as was the difference between low-practicing musicians and non-musicians (*p* = 0.304).

### Inhibition

**Stroop.** A 2 × 3 (language status [bilingual, monolingual] × musician status [high-practicing musician, low-practicing musician, non-musician]) between-subjects ANOVA did not demonstrate any significant main effects or interactions. When musician status was divided into high-practicing, low-practicing, and non-musician groups, a trend in favor of musician benefits was no longer demonstrated on the Stroop task. In addition, contrary to our predictions, there was no significant main effect of language, nor an interaction between musician and language status.

**Flanker.** Examination of performance on the task across the three levels of musician status produced results that were consistent with original findings. A 2 × 2 × 3 (congruency [congruent, incongruent] × language status [bilingual, monolingual] × musician status [high-practicing musician, low-practicing musician, non-musician]) repeated-measures ANOVA was run. Results indicated a main effect of congruency, *F*(2, 90) = 150.81, *p* < 0.001, η^2^ = 0.626, with faster performance on congruent (mean = 487.9, SEM = 7.6) vs incongruent (mean = 539.8, SEM = 7.7) trials. No musician or language benefits were found in this analysis. However, tests of between-subjects effects demonstrated a marginal main effect of music, *F*(2, 90) = 2.62, *p* = 0.079, η^2^ = 0.055, with faster performance among high-practicing musicians (mean = 498.5, SEM = 14.3) than low-practicing musicians (mean = 508.1, SEM = 13.5) or non-musicians (mean = 535.0, SEM = 10.1).

**Stop signal.** Examination of musician status at three levels produced results that were consistent with initial findings. That is, results of a 2 × 3 (language status [bilingual, monolingual] × musician status [high-practicing musician, low-practicing musician, non-musician]) between-subjects ANOVA for the stop signal task did not demonstrate any significant main effects for musician or language status, and no music by language status interaction was found (*p*s > 28). Sample size limitations did not allow us to further break down this group into musicians who were part of an orchestra or band, as we had in the original analyses.

## Appendix 3

### Training classification re-analysis

Further analyses using more stringent classification criteria for bilingualism and musician status were conducted in order to establish that our sample selection criteria for the present study was comparable to that of other literature examining bilinguals and musicians (Bidelman et al., 2013; Paap & Greenberg, 2013). Our revised classification criteria included bilinguals who considered themselves completely fluent in both languages and used both languages on a daily basis, while musicians consisted of instrumentalists who played their instrument regularly, who had at least ten years of training beginning at or before the age of 13 years and had at least five years of formal lessons. A total of 52 participants did not meet these criteria and were removed from the analysis, leaving data for 100 participants to be analyzed. However, no change was seen in the pattern of results.

### Analyses

#### Working memory

**Digit span.** Consistent with the previous findings for the digit span backward task, the results of a 2 × 2 (musician status [musician, non-musician] × language status [bilingual, monolingual]) between-subjects ANOVA showed a main effect of musician status, *F*(3, 96) = 7.68, *p* = 0.007, η^2^ = 0.074, with musicians (*M*_*mus*_ = 8.97) outperforming non-musicians (*M*_*n-mus*_ = 7.44) on the digit span backward. Similar to before, there was neither a main effect of language status, nor an interaction between musician and language status (*p*s > 0.58). However, the results of the follow-up analysis were not consistent with that of the original analysis for digit span forward. A musician benefit and a monolingual advantage, which had been demonstrated earlier were no longer present. Here, results may have been affected by low power when the sample was reduced. However, data patterns and effect sizes were consistent with the original analysis.

**Reading span.** A 2 × 2 (musician status [musician, non-musician] × language status [bilingual, monolingual]) between-subjects ANOVA demonstrated a significant interaction between musician and language status, *F*(3, 95) = 4.28, *p* = 0.041, η^2^ = 0.043. A follow up *t-*test demonstrated higher accuracy in monolingual musicians (mean = 57.4, SD = 9.8) compared to monolingual non-musicians (mean = 47.5, SD = 11.7) on this task, *t*(68) = 3.81, *p* < 0.001. However, a significant difference in accuracy between bilingual musicians and bilingual non-musicians was not detected (*p* > 0.78). No main effects of music or language status were demonstrated.

**Operation span.** The results of a 2 × 2 (musician status [musician, non-musician] × language status [bilingual, monolingual]) between-subjects ANOVA demonstrated a main effect of musician status, *F*(3, 31) = 5.97, *p* = 0.02, η^2^ = 0.161, with musicians (*M*_*mus*_ = 61.9, SEM = 3.7) outperforming non-musicians (*M*_*n-mus*_ = 49.5, SEM = 3.4) on the operation span. Neither a main effect of language status, nor a music by language status interaction was found.

#### Inhibition

**Stroop.** A 2 × 2 (musician status [musician, non-musician] × language status [bilingual, monolingual]) between-subjects ANOVA demonstrated a marginal main effect of music, *F*(3, 95) = 3.34, *p* = 0.07, η^2^ = 0.034, with musicians (*M*_*mus*_ = 12.3, SEM = 1.1) outperforming non-musicians (*M*_*n-mus*_ = 14.9, SEM = 0.9) in terms of reaction time (in seconds) on the stroop task. A main effect of language or a music by language status interaction was not demonstrated in this analysis (*ps* > 0.29).

**Flanker.** Results of a 2 × 2 × 2 (musician status [musician, non-musician] × language status [bilingual, monolingual] × congruency [incongruent, congruent]) repeated measures ANOVA demonstrated a main effect of congruency, *F*(3, 60) = 147.13, *p* < 0.001, η^2^ = 0.710, with faster performance on congruent (mean = 485, SEM = 8.5) than incongruent (mean = 540, SEM = 8.3) trials. No musician or language benefits were found in this analysis. However, tests of between-subjects effects demonstrated a main effect of music, *F*(3, 60) = 15.88, *p* < 0.001, η^2^ = 0.209, with faster performance among musicians (mean = 480, SEM = 11.7) than non-musicians (mean = 545, SEM = 11.1). Moreover, an independent samples *t*-test examining performance of musicians and non-musicians on the Flanker task revealed significant differences in RT on all conditions, including null (*t*(62) = − 3.52, *p* = 0.001), neutral (*t*(62) = − 3.18, *p* = 0.002), congruent (*t*(62) = − 3.79, *p* < 0.001), and incongruent (*t*(62) = − 4.74, *p* < 0.001) trials. The findings for this analysis were consistent with that of the original Flanker analysis.

**Stop signal.** A 2 × 2 (musician status [musician, non-musician] × language status [bilingual, monolingual]) between-subjects ANOVA was performed on the no signal reaction time component of the stop signal task. Consistent with earlier analyses, the results demonstrated neither a music or language main effect, nor a music by language status interaction (*p*s > 0.17).

## Footnotes

1 Separate publications were created to separate conceptually independent hypotheses, results, and findings.

2 We chose to use a stepwise Bonferroni-type procedure for multiple testing since simulation studies have confirmed that such procedures generally perform as well or better than more complicated alternative procedures (Cribbie, 2017). A family-wise alpha correction was used per construct. Many researchers advocate against using experiment-wise alpha corrections as it is overly conservative and often not conceptually meaningful, especially in situations such as our study when the general null hypothesis of no significant effects across all tasks is not primarily of interest (review in Cribbie, 2017). However, if we were to use an experiment-wise alpha correction, only one of our results would change. The operation span task would no longer be significant, since obtained *p* value, *p* = 0.018, fails to meet the cutoff *p* = 0.017. However, it misses the cutoff by 0.01 decimal place and we still find a significant effect on two out of three working memory measures.
